# Genome sequence and description of *Gracilibacillus timonensis* sp. nov. strain Marseille‐P2481^T^, a moderate halophilic bacterium isolated from the human gut microflora

**DOI:** 10.1002/mbo3.638

**Published:** 2018-04-19

**Authors:** Awa Diop, El hadji Seck, Gregory Dubourg, Nicholas Armstrong, Caroline Blanc‐Tailleur, Didier Raoult, Pierre‐Edouard Fournier

**Affiliations:** ^1^ URMITE, UM63, CNRS 7278, IRD 198, Inserm U1095 Aix‐Marseille Université Institut hospitalo‐universitaire Mediterranee‐infection Marseille France; ^2^ Special Infectious Agents Unit King Fahd Medical Research Center King Abdulaziz University Jeddah Saudi Arabia

**Keywords:** *Gracilibacillus timonensis*, halophilic, human gut flora, microbial culturomics, taxonogenomics

## Abstract

Microbial culturomics represents an ongoing revolution in the characterization of the human gut microbiota. By using three culture media containing high salt concentrations (10, 15, and 20% [w/v] NaCl), we attempted an exhaustive exploration of the halophilic microbial diversity of the human gut and isolated strain Marseille‐P2481 (= CSUR P2481 =  DSM 103076), a new moderately halophilic bacterium. This bacterium is a Gram‐positive, strictly aerobic, spore‐forming rod that is motile by use of a flagellum and exhibits catalase, but not oxidase activity. Strain Marseille‐P2481 was cultivated in media containing up to 20% (w/v) NaCl, with optimal growth being obtained at 37°C, pH 7.0–8.0, and 7.5% [w/v] NaCl). The major fatty acids were 12‐methyl‐tetradecanoic acid and hexadecanoic acid. Its draft genome is 4,548,390 bp long, composed of 11 scaffolds, with a G+C content of 39.8%. It contains 4,335 predicted genes (4,266 protein coding including 89 pseudogenes and 69 RNA genes). Strain Marseille‐P2481 showed 96.57% 16S rRNA sequence similarity with *Gracilibacillus alcaliphilus* strain SG103^T^, the phylogenetically closest species with standing in nomenclature. On the basis of its specific features, strain Marseille‐P2481^T^ was classified as type strain of a new species within the genus *Gracilibacillus* for which the name *Gracilibacillus timonensis* sp. nov. is formally proposed.

## 1 INTRODUCTION

One of the most important methods of food preservation in history has been the use of salt (NaCl). Salt has also become an indispensable ingredient of any kitchen. Considered previously as hostile to most forms of life by limiting the growth of certain bacteria, it was demonstrated to favor the emergence and growth of others, mainly halophilic bacteria (Cantrell, Dianese, Fell, Gunde‐Cimerman, & Zalar, 2011). Several recent studies have reported the isolation of new halophilic species from the human gut microflora (Khelaifia et al., 2016; Lagier, Khelaifia, et al., 2015). Therefore, exploring the diversity of halophilic microorganisms in the human gut flora may provide important insights into our understanding of their presence, interactions with the human digestive environment, and their influence on health.

In order to explore the human gut halophilic microbiota, and as part of the ongoing microbial culturomics study in our laboratory (Lagier et al., 2012, 2016), we used high salt‐containing culture media, which enabled us to isolate a new moderately halophilic bacterial strain, Marseille‐P2481, that belongs to the genus *Gracilibacillus* (Senghor et al., 2017). First proposed by Wainø et al. in 1999 (Wainø, Tindall, Schumann, & Ingvorsen, 1999), the genus *Gracilibacillus* currently includes 13 species ( http://www.bacterio.net/gracilibacillus.html) with validly published names (Parte, 2014). These are Gram stain‐positive, aerobic, moderately halophilic or halotolerant, motile bacteria. In most species, cells are motile due to peritrichous flagella and form endospores and white colonies (Wainø et al., 1999). *Gracilibacillus* species were isolated from diverse salty environmental samples, including sea water, salty lakes (Gao et al., 2012; Jeon et al., 2008), soil (Chen et al., 2008; Huo, Xu, Cui, & Wu, 2010), and/or food (Chamroensaksri et al., 2010; Diop et al., 2016).

Using the taxonogenomics approach that includes phenotypic features, proteomic information obtained by matrix‐assisted laser‐desorption/ionization time‐of‐flight mass spectrometry (MALDI‐TOF MS), and analysis of the complete genome sequence (Pagani et al., 2012; Ramasamy et al., 2014; Sentausa & Fournier, 2013), we present here the characterization of a new halophilic species for which we formally propose the name *Gracilibacillus timonensis* sp. nov. Strain Marseille‐P2481^T^ (= CSUR P2481 =  DSM 103076) is the type strain of *Gracilibacillus timonensis* sp. nov.

## 2 MATERIALS AND METHODS

### 2.1 Sample collection and culture conditions

A stool sample was collected from a 10‐year‐old healthy young Senegalese boy living in N'diop (a rural village in the Guinean‐Sudanian zone of Senegal). The patient's parents gave an informed consent, and the study was approved by the National Ethics Committee of Senegal (N° 00.87 MSP/DS/CNERS) and by the local ethics committee of the IFR48 (Marseille, France) under agreement 09‐022. The stool sample was collected immediately after defecation into a sterile plastic container, preserved at −80°C and transported to Marseille until further analysis.

The salinity of the sample was measured using a digital refractometer (Fisher scientific, Illkirch, France) and its pH measured using a pH‐meter (Eutech Instruments, Strasbourg, France).

Strain Marseille‐P2481 was isolated in aerobic conditions, on a home‐made culture medium consisting of Columbia agar enriched with 10% (w/v) NaCl (Sigma‐Aldrich, Saint‐Louis, MO, USA), as previously described (Diop et al., 2016). Briefly, 1 g of stool sample was inoculated into 100 ml of our home‐made liquid medium and incubated aerobically at 37°C. Subcultures were conducted after 1, 3, 7, 10, 15, 20, and 30 days of incubation. Serial dilutions of 10^−1^ to 10^−10^ were then performed in the home‐made liquid culture medium and plated on Columbia and Chapman agar plates (Oxoid, Dardilly, France). After 2 days of incubation at 37°C, all apparent colonies were picked and subcultured several times to obtain pure cultures.

### 2.2 MALDI‐TOF MS strain identification

Briefly, one isolated bacterial colony was picked from chapman culture plate using a pipette tip and spread it as a thin film on a MTP 96 MALDI‐TOF target plate for identification with a Microflex MALDI‐TOF MS spectrometer (Bruker Daltonics, Leipzig, Germany). In total, 12 distinct deposits for strain Marseille‐P2481were done from 12 individual colonies in duplicate. After air‐drying, 2‐μl matrix solution was applied per spot, as previously reported (Lagier, Khelaifia, et al., 2015). All spectra were recorded in positive linear mode for the mass range of 2,000–20,000 Da (parameter settings: ion source 1 (ISI), 20 kV; IS2, 18.5 kV; lens, 7 kV). The obtained protein spectra were compared with those of 2,480 spectra in the Bruker database enriched with our own database (Lagier, Hugon, et al., 2015). The strain was identified at the species level if the MALDI‐TOF MS score was greater than 1.9. If the score was lower than this threshold, the identification was not considered as reliable and the 16S rRNA gene was sequenced.

### 2.3 16S rRNA gene sequencing identification

The 16S rRNA gene was amplified using the broad‐range primer pair FD1 and rp2 (Drancourt et al., 2000). The primers were obtained from Eurogentec (Seraing, France). The obtained amplicon was sequenced using the Big Dye Terminator Sequencing kit and the following internal primers: 536f, 536r, 800f, 800r, 1050f, 1050r, 357f, 357r, as previously described (Drancourt, Bollet, & Raoult, 1997; Drancourt et al., 2000). The sequence was then compared with the NCBI database using the BLASTn algorithm ( https://blast.ncbi.nlm.nih.gov/). If the 16S rRNA gene sequence similarity value was greater than 95% and lower 98.65% with the most closely related species with standing in nomenclature, as previously proposed (Kim, Oh, Park, & Chun, 2014; Stackebrandt & Ebers, 2006), the strain was proposed to belong to a new species (Konstantinidis, Ramette, & Tiedje, 2006).

### 2.4 Phylogenetic analysis

The 16S sequences from the type strains of the species with a validly published name that exhibited the highest BLAST score with our new strain were downloaded from the NCBI ftp server ( ftp://ftp.ncbi.nih.gov/Genome/). Sequences were aligned using the CLUSTALW 2.0 software (Larkin et al., 2007), and phylogenetic inferences were obtained using the neighbor‐joining method and the maximum likelihood method within the MEGA software, version 6 (Tamura, Stecher, Peterson, Filipski, & Kumar, 2013). The evolutionary distances were computed based on the Kimura 2‐parameter model (Kimura, 1980) with 95% of deletion, and bootstraping analysis was performed with 500 replications.

### 2.5 Morphological observation

To observe the cell morphology, transmission electron microscopy of the strain was performed using a Tecnai G20 Cryo (FEI company, Limeil‐Brevannes, France) at an operating voltage of 60 Kv after negative staining. Gram staining was performed and observed using a photonic microscope Leica DM2500 (Leica Microsystems, Nanterre, France) with a 100X oil‐immersion objective (Atlas & Snyder, 2011). The motility of the strain was assessed by the Hanging Drop method. The slide was examined using a DM1000 photonic microscope (Leica Microsystems) at 40×. Sporulation was tested following a thermic shock at 80°C during 20 min, and the endospore formation was visualized using a Tecnai G20 Cryo transmission electron microscope (FEI company, Limeil‐Brevannes, France) at an operating voltage of 60 Kv after negative staining.

### 2.6 Atmospheric tests, biochemical, and antimicrobial susceptibility

In order to evaluate the optimal culture conditions, strain Marseille‐P2481 was cultivated on Chapman agar at different temperatures (25, 28, 37, 45 and 56°C) under aerobic conditions, and in anaerobic and microaerophilic atmospheres using GENbag Anaer and GENbag microaer systems (bioMérieux), respectively. The pH (pH 5, 6, 6.5, 7, and 8.5) and salinity (5–20% [w/v] NaCl) conditions were also tested.

Biochemical tests were performed using the API ZYM, API 50 CH, and API 20 NE strips (bioMerieux, Marcy‐l'Etoile, France), according to the manufacturer's instructions. The API ZYM was incubated for 4 hr and the other two strips for 48 hr.

The antibiotic susceptibility of strain Marseille‐P2481 was determined using the disk diffusion method as previously described (Diop et al., 2016). The following antibiotics were tested: penicillin G **(**10 μg), amoxicillin (25 μg), ceftriaxone (30 μg), imipenem (10 μg), rifampicin (30 μg), erythromycin (15 μg), gentamicin (500 μg), and metronidazole (4 μg). The results were interpreted using the Scan 1,200 automate (Interscience, Saint Nom la Bretêche, France).

### 2.7 Fatty acid methyl ester (FAME) analysis by GC/MS

For the FAME analysis, strain Marseille‐P2481 was cultivated on Chapman agar (7.5% NaCl) (Oxoid, Dardilly, France) at 37°C under aerobic atmosphere for 2 days. Cellular fatty acid methyl ester (FAME) analysis was performed by gas chromatography/mass spectrometry (GC/MS). Two samples were prepared with approximately 70 mg of bacterial biomass per tube harvested from several culture plates. FAMEs were prepared as described by Sasser (Sasser, 1990). GC/MS analyses were carried out as previously described (Dione et al., 2016). Briefly, FAMEs were separated using an Elite 5‐MS column and monitored by mass spectrometry (Clarus 500 ‐ SQ 8 S, Perkin Elmer, Courtaboeuf, France). Spectral database search was performed using the MS Search 2.0 software operated with the Standard Reference Database 1A (NIST, Gaithersburg, USA) and the FAMEs mass spectral database (Wiley, Chichester, UK).

### 2.8 Extraction and genome sequencing

After a pretreatment by lysozyme incubation at 37°C for 2 hr, the DNA of strain Marseille‐P2481 was extracted on the EZ1 biorobot (Qiagen) with EZ1 DNA Tissue kit. The elution volume was 50 μl. The gDNA was quantified by a Qubit assay with the high sensitivity kit (Life Technologies, Carlsbad, CA, USA) to 185 ng/μl.

A MiSeq sequencer and the mate‐pair strategy (Illumina Inc, San Diego, CA, USA) were used to sequence the gDNA. The gDNA was barcoded in order to be mixed with 11 other projects with the Nextera Mate‐Pair sample prep kit (Illumina). The mate‐pair library was prepared with 1.5 μg of gDNA using the Nextera mate‐pair guide. The genomic DNA sample was simultaneously fragmented and tagged with a mate‐pair junction adapter. The pattern of the fragmentation was validated on an Agilent 2100 BioAnalyzer (Agilent Technologies Inc, Santa Clara, CA, USA) with a DNA 7500 labchip. The DNA fragments ranged in size from 1.5 to 11 kb with an optimal size at 5.314 kb. No size selection was performed and 600 ng of tagmented fragments was circularized. The circularized DNA was mechanically sheared with an optimal size at 939 bp on the Covaris device S2 in T6 tubes (Covaris, Woburn, MA, USA). The library profile was visualized on a High Sensitivity Bioanalyzer LabChip (Agilent Technologies Inc, Santa Clara, CA, USA), and the final concentration library was measured at 8.38 nmol/L. The libraries were normalized at 2 nmol/L and pooled. After a denaturation step and dilution at 15 pM, the pool of libraries was loaded. Automated cluster generation and sequencing run were performed in a single 39‐hr run in a 2 × 251 bp.

A total sequencing output of 6.52 Gb was obtained from a 696 K/mm^2^ cluster density with a cluster passing quality control filters of 95.6% (12,863,388 passing filter paired reads). Within this run, the index representation for strain Marseille‐P2481 was determined to be 9.39%. The 1,207,306 paired reads were trimmed and then assembled.

### 2.9 Genome annotation and comparison

Prodigal was used for open reading frame (ORF) prediction (Hyatt et al., 2010) with default parameters. Predicted ORFs spanning a sequencing gap region were excluded. Bacterial protein sequences were predicted using BLASTP (E‐value 1e^−03^, coverage 0.7 and identity percent 30%) against the Clusters of Orthologous Groups (COG) database. If no hit was found, a search against the nr database (Benson et al., 2015) was performed using BLASTP with E‐value of 1e^−03^, a coverage of 0.7 and an identity percent of 30%. If sequence lengths were smaller than 80 amino acids, we used an E‐value of 1e^−05^. Pfam conserved domains (PFAM‐A an PFAM‐B domains) were searched on each protein with the HHMscan tool (Finn et al., 2015). RNAmmer (Lagesen et al., 2007) and tRNAScanSE (Lowe & Eddy, 1997) were used to identify ribosomal RNAs and tRNAs, respectively. We predicted lipoprotein signal peptides and the number of transmembrane helices using Phobius (Käll, Krogh, & Sonnhammer, 2004). ORFans were identified if the BLASTP search was negative (E‐value smaller than 1e^−03^ for ORFs with a sequence size larger than 80 aas or E‐value smaller than 1e^−05^ for ORFs with sequence length smaller than 80 aas). Artemis (Carver, Harris, Berriman, Parkhill, & McQuillan, 2012) and DNA Plotter (Carver, Thomson, Bleasby, Berriman, & Parkhill, 2009) were used for data management and for visualization of genomic features, respectively. Genomes from members of the genus *Gracilibacillus* and closely related genera were used for the calculation of AGIOS values. The genome of strain Marseille‐P2481 (EMBL‐EBI accession number FLKH00000000) was compared with those of *Gracilibacillus halophilus* strain YIM‐C55.5^T^ (APML00000000), *G. boraciitolerans* strain JCM 21714^T^ (BAVS00000000), *G. lacisalsi* strain DSM 19029 ^T^ (ARIY00000000), *G. massiliensis* strain Awa‐1^T^ (CZRP00000000), *G. kekensis* strain K170 ^T^ (FRCZ01000001), *G. orientalis* strain XH‐63 ^T^ (FOTR01000001), *G. ureilyticus* strain MF38 ^T^ (FOGL01000001), *B. clausii* strain KSM‐K16^T^ (AP006627), and *B. alcalophilus* strain ATCC 27647^T^ (ALPT00000000). Annotation and comparison processes were performed using the multi‐agent software system DAGOBAH (Gouret et al., 2011), which includes Figenix (Gouret et al., 2005) libraries that provide pipeline analysis. We also estimated the degrees of genomic sequence similarity among compared genomes using the following tools: first, we used the MAGI home‐made software (Padmanabhan, Mishra, Raoult, & Fournier, 2013) This software calculates the average genomic identity of orthologous gene sequences (AGIOS) among compared genomes (Ramasamy et al., 2014). It combines the Proteinortho software (Lechner et al., 2011) for detecting orthologous proteins in pairwise genomic comparisons, then retrieves the corresponding genes and determines the mean percentage of nucleotide sequence identity among orthologous ORFs using the Needleman‐Wunsch global alignment algorithm. Second, the digital DNA–DNA hybridization was performed using the GGDC (Genome‐to‐Genome Distance Calculator) analysis via the GGDC web server as previously reported (Klenk, Meier‐Kolthoff, & Göker, 2014). Finally, the average amino acid identity (AAI) was calculated, based on the overall similarity between two genomic datasets of proteins (Rodriguez‐R & Konstantinidis, 2014) available at ( http://enve-omics.ce.gatech.edu/aai/index).

## 3 RESULTS

### 3.1 Strain identification and phylogenetic analysis

**Figure 1:**
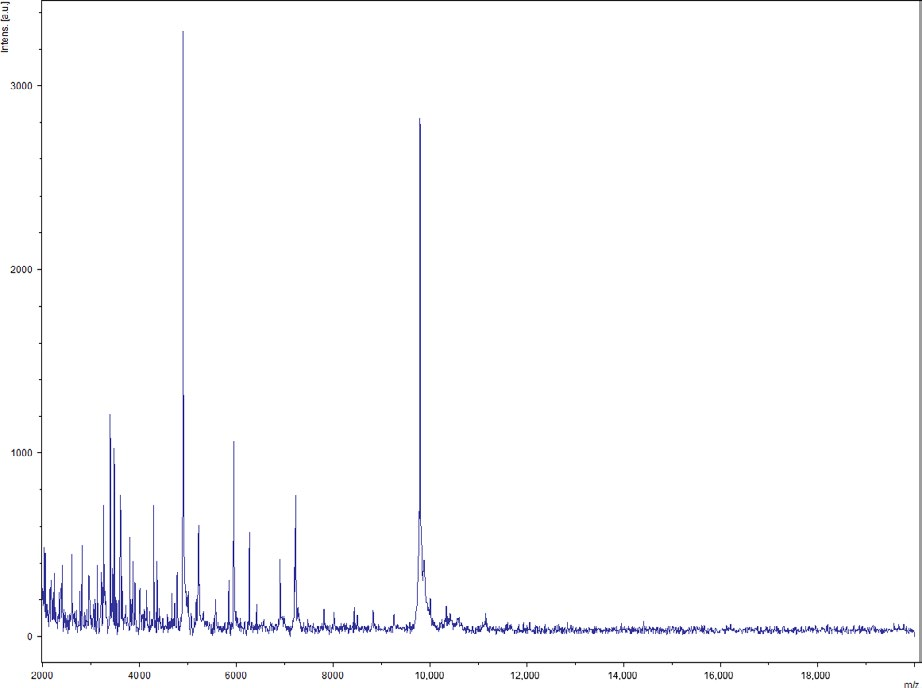
Reference mass spectrum from *Gracilibacillus timonensis* strain Marseille‐P2481^T^

**Figure 2:**
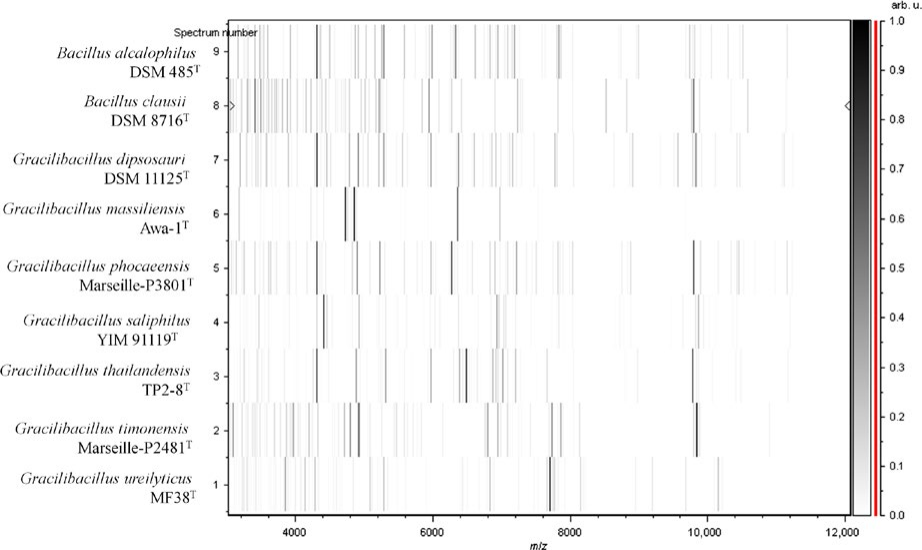
Gel view comparing *Gracilibacillus timonensis* strain Marseille‐P2481^T^ with other species within the genera *Gracilibacillus* and *Bacillus*

**Figure 3:**
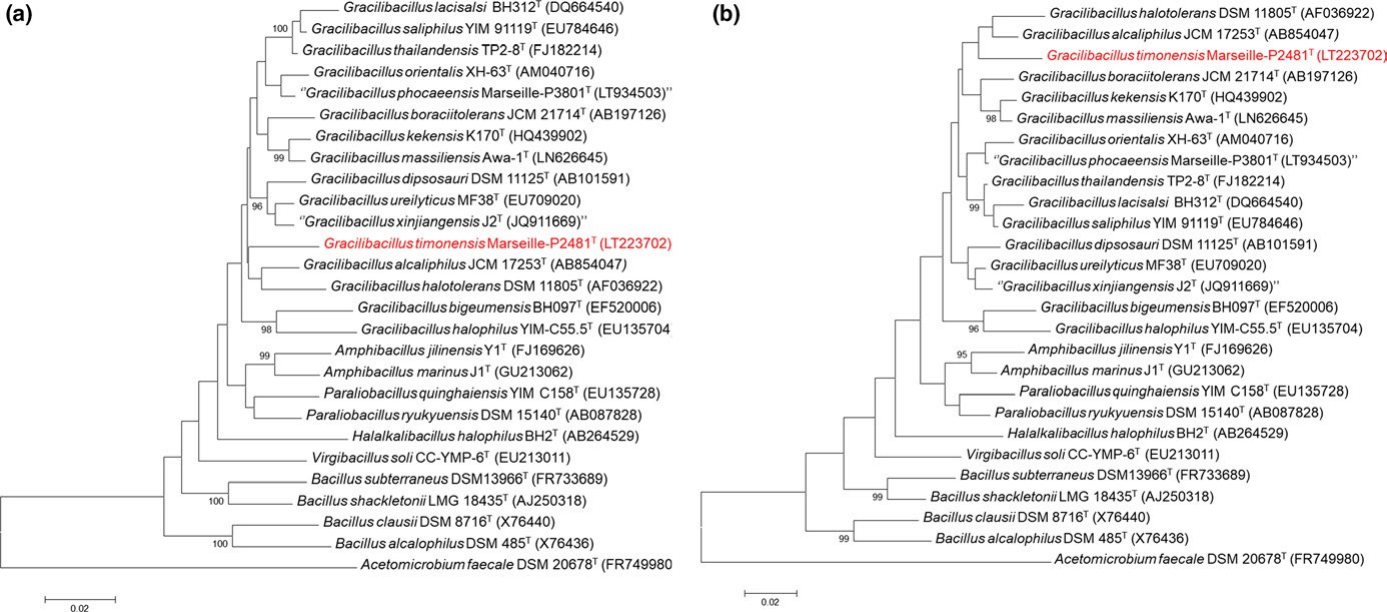
Phylogenetic tree highlighting the position of *Gracilibacillus timonensis* strain Marseille‐P2481^T^ relative to other closely related species. GenBank accession numbers of each 16S rRNA are indicated after each species name. Sequences were aligned using CLUSTALW, and the evolutionary history was inferred using the Neighbor‐Joining method (a) and the maximum likelihood method (b) with the Kimura 2‐parameter method within MEGA6 software. The percentage of replicate trees in which the associated taxa clustered together in the bootstrap test (500 replicates) is shown next to the branches. The analysis involved 24 nucleotide sequences. All positions with less than 95% site coverage were eliminated. There were a total of 1,404 positions in the final dataset. The scale bar represents a 2% nucleotide sequence divergence

A MALDI‐TOF MS score of 1.4 was obtained for strain Marseille‐P2481 against our database (Bruker database), suggesting that our isolate was not in the database. The MALDI‐TOF MS spectrum from strain Marseille‐P2481 (Figure 1) was added to our database and a gel view showed the spectral differences between our isolate and other closely related species (Figure 2). The 16S rDNA‐based identification of strain Marseille‐P2481 (EMBL‐EBI accession number LT223702) yielded a 96.57% 16S rRNA gene sequence identity with *Gracilibacillus alcaliphilus* strain SG103^T^ (GenBank accession number NR_126185), the phylogenetically closest species with a validly published name (Figure 3). As this value was lower than the 98.65% 16S rRNA sequence identity threshold recommended to define a new species without carrying out DNA–DNA hybridization (Kim et al., 2014), strain Marseille‐P2481 was considered as representative of a potential new species within the *Gracilibacillus* genus.

### 3.2 Physiological and biochemical characteristics

**Figure 4:**
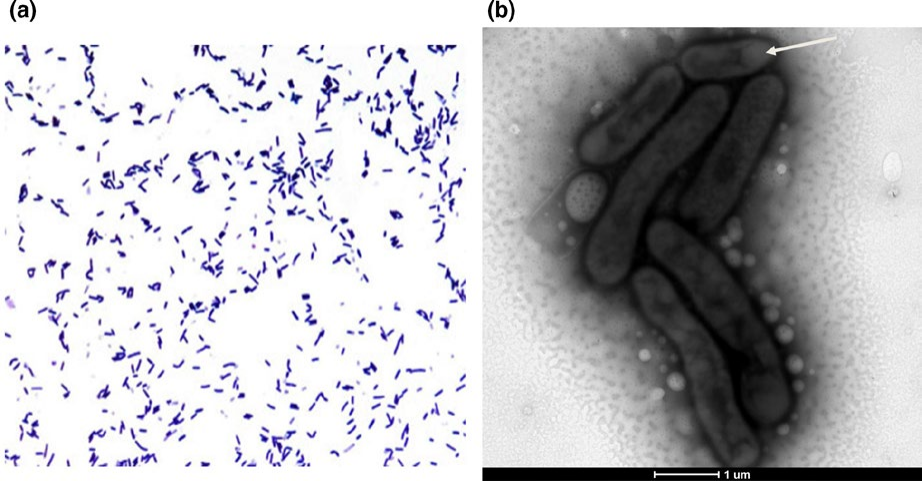
Bacterial morphology. (a) Gram staining of *Gracilibacillus timonensis* strain Marseille‐P2481^T^. (b) Transmission electron microscopy showing an endospore in terminal position (arrow). The scale bar represents 1 μm

Isolated for the first time in our home‐made halophilic medium with 10% (w/v) NaCl, strain Marseille‐P2481 was able to grow in media containing up to 20% (w/v) NaCl under aerobic conditions with a minimal concentration of growth at 7.5% NaCl, but was also able to grow in anaerobic and microaerophilic atmospheres (at 37°C). After 2 days of growth at 37°C, colonies were creamy orange and circular, with a mean diameter of 0.2 μm. Cells were Gram stain‐positive (Figure 4a), endospore‐forming (Figure 4b), and motile rods with a peritrichous flagellum. Cells were also slightly curved, with mean diameter and length of 0.5 and 1.9 μm, respectively (Figure 4b). Strain Marseille‐P2481 exhibited positive catalase but no oxidase activity. General features and classification of *Gracilibacillus timonensis* strain Marseille‐P2481^T^ are summarized in Table 1. Using an API ZYM strip, positive results were obtained for esterase, esterase lipase, acid phosphatase, naphtol‐AS‐BI‐phosphohydrolase β‐galactosidase, β‐glucosidase, and α‐glucosidase activities but no reaction was observed for alkaline phosphatase, lipase, Leucine arylamidase, Valine arylamidase, Cystine arylamidase, α‐galactosidase, β‐glucuronidase, trypsin, α‐chymotrypsin, α‐mannosidase, α‐fucosidase, and N‐acetyl‐β‐glucosaminidase. The API 50CH strip revealed that strain Marseille‐P2481 exhibited esculin hydrolysis, but negative reactions were obtained for d‐arabitol, l‐arabitol, d‐glucose, d‐fructose, d‐fucose, d‐galactose, d‐lactose, d‐maltose, d‐ribose, d‐saccharose, d‐lyxose, d‐mannose l‐sorbose, d‐tagatose, d‐turanose, d‐xylose, l‐xylose, d‐arabinose, l‐arabinose, d‐sorbitol, d‐cellobiose, d‐melezitose, d‐melibiose, d‐trehalose, d‐raffinose, l‐rhamnose, d‐adonitol, d‐mannitol, l‐fucose, amygdalin, arbutin, erythritol, dulcitol, gentiobiose, glycerol, glycogen, inositol, inulin, salicin, starch, xylitol, αD‐glucopyranoside, methyl‐βD‐xylopyranoside, methyl‐αD‐mannopyranoside, potassium gluconate, potassium‐2‐ketogluconate potassium‐5‐ketogluconate, N‐acetylglucosamine. Using an API 20NE strip, fermentation of glucose, urease activity, and metabolism of l‐arginine, esculin and 4‐nitrophenyl‐βD‐galactopyrasinoside were positive. In contrast, nitrate and indole production, gelatinase activity and metabolism of d‐glucose, l‐arabinose, d‐mannose, d‐maltose, d‐mannitol, N‐acetyl‐glucosamine, potassium gluconate, capric acid, malic acid, trisodium citrate, and phenylacetic acid were negative. Strain Marseille‐P2481 differed from all other studied members of the genus *Gracilibacillus* in a combination of negative alkaline phosphatase and nitrate reductase activities but the acidification of d‐fructose (Table 2). The cellular fatty acids from strain Marseille‐P2481 are mainly saturated and the most abundant were 12‐methyl‐tetradecanoic acid, hexadecanoic acid, and 14 methyl‐hexadecanoic acid (45%, 16%, and 14%, respectively). No unsaturated fatty acid was detected (Table 3). Cells are resistant to Penicillin G, amoxicillin, ceftriaxone, and metronidazole, but susceptible to imipenem, rifampicin, gentamicin, and erythromycin.

**Table 1:** Classification and general features of *Gracilibacillus timonensis* strain Marseille‐P2481^T^ according to the MIGS recommendations [23]

MIGS ID	Property	Term	Evidence code^a^
		Domain: *Bacteria*	TAS (Woese, Kandler, & Wheelis, 1990)
		Phylum: *Firmicutes*	TAS (Skerman & Sneath 1980, Murray, 1984, Gibbons and Murray, 1978, Garrity and Holt, 2001)
		Class: *Bacilli*	TAS (Ludwig, Schleifer, & Whitman 2009)
		Order: *Bacillales*	TAS (Skerman & Sneath 1980, Prevot, 1953)
		Family: *Bacillaceae*	TAS (Skerman & Sneath 1980, Fischer, 1895)
		Genus: *Gracilibacillus*	TAS (Wainø et al., 1999)
		Species: *Gracilibacillus timonensis*	IDA
		Type strain: Marseille‐P2481^T^	IDA
	Gram stain	Positive	IDA
	Cell shape	Rods	IDA
	Motility	Motile	IDA
	Sporulation	Spore‐forming	IDA
	Temperature (°C)	Mesophile (25‐45)	IDA
	Optimum temperature	37°C	IDA
	pH range: Optimal pH	6.0–9.0 7.0–8.0	IDA
	Carbon source	Unknown	IDA
MIGS‐6	Habitat	Human gut	IDA
MIGS‐6.3	NaCl range: Optimum NaCl	7.5–20% 7.5%	IDA
MIGS‐22	Oxygen requirement	Aerobic	IDA
MIGS‐15	Biotic relationship	Free living	IDA
MIGS‐14	Pathogenicity	Unknown	IDA

a Evidence codes: IDA, Inferred from Direct Assay; TAS, Traceable Author Statement (i.e., a direct report exists in the literature). These evidence codes are from the Gene Ontology project (Ashburner et al. 2000).

**Table 2:** Differential characteristics of *Gracilibacillus timonensis* strain Marseille‐P2481^T^ and other closely related members of the genus *Gracilibacillus*

Properties	*G. timonensis*	*G. saliphilus*	*G*. *bigeumensis*	*G. halophilus*	*G. boraciitolerans*	*G. kekensis*	*G. halotolerans*	*G. alcaliphilus*
Cell diameter (μm)	0.5–0.8	0.7–0. 9	0.3–0.5	0.3–0.5	0.5–0. 9	0.2–1.05	0.4–0.6	0.5–0.7
Pigmentation	Creamy orange	Creamy white	Creamy	White	Dirty white	Creamy white	Creamy white	Creamy white
Oxygen requirement	Aerobic	Aerobic	Aerobic	Aerobic	Aerobic	Aerobic	Aerobic	Aerobic
Gram stain	+	+	+	+	+	+	+	+
Salt requirement	+	+	+	+	+	+	+	+
Motility	+	+	+	+	+	+	+	+
Sporulation	+	+	+	+	+	+	**+**	+
Indole	−	−	−	−	−	−	−	−
Production of								
Alkaline phosphate	−	+	+	+	+	NA	+	−
Catalase	+	+	+	+	+	NA	+	+
Oxidase	−	+	+	+	+	−	+	−
Nitrate reductase	−	+	−	+	−	−	+	+
Urease	+	+	−	−	−	−	+	+
β‐galactosidase	+	+	+	+	+	NA	−	NA
α‐galactosidase	−	−	−	−	+	NA	+	−
N‐acetyl‐glucosamine	−	+	−	−	NA	NA	NA	+
l‐arabinose	−	+	+	−	+	+	+	+
Ribose	−	+	−	+	+	+	+	+
d‐mannose	−	+	+	−	+	+	−	−
d‐mannitol	−	+	+	+	+	+	+	+
d‐glucose	+	+	+	+	+	+	+	+
d‐fructose	−	+	+	+	+	+	+	+
d‐maltose	−	+	+	−	+	+	−	+
d‐lactose	−	+	**+**	−	+	+	−	+
DNA G+C content (mol %)	39.8	40.1	37.9	42.3	35.8	35.8	38	41.3
Habitat	Human gut	Salt lake	Solar saltern soil	Salty soil	Soil	Salty lake	Saline soil	Fermentation liquor for dyeing

NA, no data available.

*G. timonensis* strain Marseille‐P2481^T^ *, G. Gracilibacillus bigeumensis* strain BH097^T^ (Kim et al., 2012)*, G. halophilus* strain YIM‐C55.5^T^ (Chen et al., 2008)*, G. boraciitolerans* strain T‐16X^T^ (Ahmed et al., 2007)*, G. saliphilus* strain YIM91119^T^ (Tang et al., 2009)*, G. kekensis* strain K170^T^ (Gao et al., 2012)*, G. halotolerans* strain NN^T^ (Wainø et al., 1999)*, G. alcaliphilus* strain SG103^T^ (Hirota, Hanaoka, Nodasaka, & Yumoto, 2014).

**Table 3:** Total cellular fatty acid composition of *Gracilibacillus timonensis* strain Marseille‐P2481^T^

Fatty acids	IUPAC name	Mean relative %^a^
15:0 anteiso	12‐methyl‐tetradecanoic acid	45.4 ± 1.5
16:0	Hexadecanoic acid	15.6 ± 1.1
17:0 anteiso	14‐methyl‐Hexadecanoic acid	13.9 ± 0.6
15:0 iso	13‐methyl‐tetradecanoic acid	10.3 ± 0.6
17:0 iso	15‐methyl‐Hexadecanoic acid	5.8 ± 1.0
16:0 iso	13‐methyl‐Pentadecanoic acid	3.4 ± 0.4
18:0	Octadecanoic acid	1.2 ± 0.1
15:0	Pentadecanoic acid	1.1 ± 0.2
14:0 iso	12‐methyl‐Tridecanoic acid	1.1 ± 0.1
17:0	Heptadecanoic acid	1.1 ± 0.1
14:0	Tetradecanoic acid	TR
10:0	Decanoic acid	TR
12:0	Dodecanoic acid	TR
13:0 anteiso	10‐methyl‐Dodecanoic acid	TR
13:0 iso	11‐methyl‐Dodecanoic acid	TR

a Mean peak area percentage calculated from the analysis of FAMEs in 2 sample preparations ± standard deviation (*n* = 3); TR= trace amounts < 1%.

### 3.3 Genome properties

The genome is 4,548,390 bp long with a 39.8% G+C content. It is composed of 11 scaffolds (composed of 12 contigs). Of the 4,335 predicted genes, 4,266 were protein‐coding genes and 69 were RNAs (4 complete 16S rRNA, 6 complete 5S rRNA gene, 2 complete and 2 partiel 23S rRNA, and 51 tRNA genes, as well as additional 4 other rRNAs). A total of 3,043 genes (70.24%) were assigned a putative function (by COGs or BLAST against nr). A total of 214 genes were identified as ORFans (6.94%). The remaining genes were annotated as hypothetical proteins (861 genes => 19.92%). The genome statistics are presented in Table 4, and the distribution of genes into COGs functional categories is summarized in Table 5.

**Table 4:** Nucleotide content and gene count of the genome

Attribute	Value	% of total^a^
Size (bp)	4,548,390	100%
G+C content (bp)	1,808,751	39.8%
Coding region (bp)	3,844,022	85.07%
Total genes	4,395	100%
RNA genes	63	1.76%
Protein‐coding genes	4,332	98.23%
Genes with function prediction	3,043	68.95%
Genes assigned to COGs	2,797	63.94%
Genes with peptide signals	474	11.20%
Genes with transmembrane helices	1,191	27.68%

a The total is based on either the size of the genome in base pairs or the total number of protein‐coding genes in the annotated genome.

**Table 5:** Number of genes associated with the 25 general COG functional categories

Code	Value	% value	Description
J	212	4.89	Translation
A	0	0	RNA processing and modification
K	266	6.14	Transcription
L	103	2.37	Replication, recombination, and repair
B	1	0.02	Chromatin structure and dynamics
D	52	1.20	Cell cycle control, mitosis, and meiosis
Y	0	0	Nuclear structure
V	98	2.26	Defense mechanisms
T	154	3.46	Signal transduction mechanisms
M	147	3.39	Cell wall/membrane biogenesis
N	49	1.13	Cell motility
Z	0	0	Cytoskeleton
W	3	0.06	Extracellular structures
U	30	0.69	Intracellular trafficking and secretion
O	107	2.46	Posttranslational modification, protein turnover, chaperones
X	57	1.31	Mobilome: prophages, transposons
C	113	2.60	Energy production and conversion
G	478	11.03	Carbohydrate transport and metabolism
E	201	4.63	Amino acid transport and metabolism
F	100	2.30	Nucleotide transport and metabolism
H	138	3.18	Coenzyme transport and metabolism
I	94	2.16	Lipid transport and metabolism
P	192	4.43	Inorganic ion transport and metabolism
Q	66	1.52	Secondary metabolites biosynthesis, transport, and catabolism
R	288	6.64	General function prediction only
S	212	4.89	Function unknown
‐	1,535	35.43	Not in COGs

### 3.4 Comparative genomics

**Figure 5:**
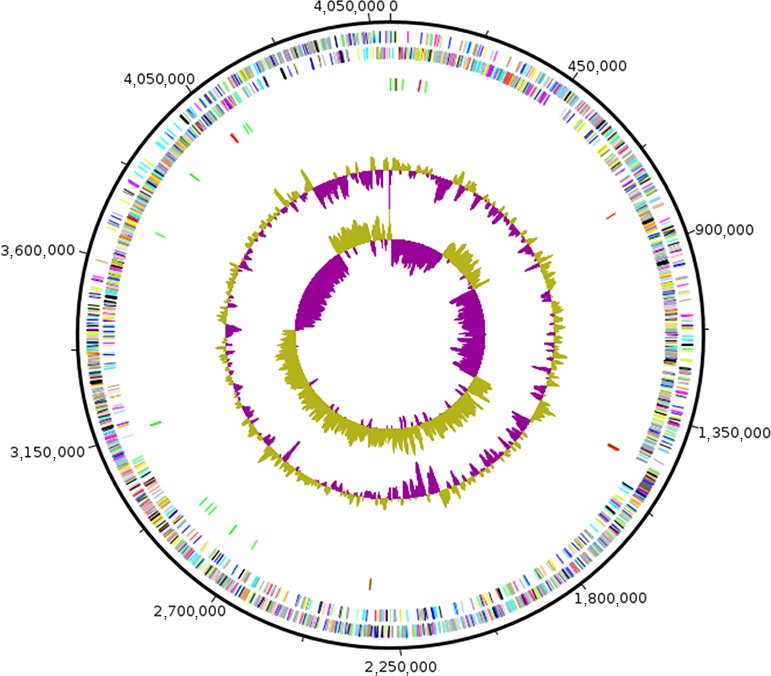
Graphical circular map of the chromosome. From the outside to the center: Genes on the forward strand colored by Clusters of Orthologous Groups of proteins (COG) categories (only genes assigned to COG), genes on the reverse strand colored by COG categories (only gene assigned to COG), RNA genes (tRNAs green, rRNAs red), GC content, and GC skew

**Figure 6:**
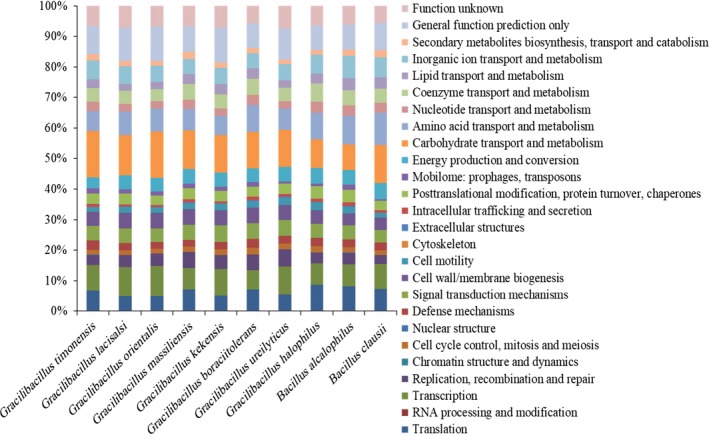
Distribution of functional classes of predicted genes according to the clusters of orthologous groups of proteins of *Gracilibacillus timonensis* strain Marseille‐P2481^T^ and other compared species

The draft genome sequence structure of strain Marseille‐P2481 is summarized in Figure 5. It is smaller than those of *G. orientalis* (4.54 and 4.61 Mb, respectively), but larger than those of G*. halophilus*,* G. boraciitolerans, G. kekensis, G. ureilyticus, G. massiliensis, B. alcalophilus, G. lacisalsi*, and *B. clausii* (3.03, 3.65, 3.93, 4.07, 4.21, 4.37, 4.41 and 4.52 Mb, respectively). The G+C content of strain Marseille‐P2481 is smaller than those of *B. clausii* (39.8 and 44.75%, respectively), but larger than those of *G. boraciitolerans, G. kekensis, G. massiliensis, G. orientalis, G. lacisalsi, B. alcalophilus, G. ureilyticus,* and G*. halophilus* (35.8, 36.0, 36.1, 36.3, 36.8, 37.4, 37.5, and 37.9%, respectively). The gene content of strain Marseille‐P2481 is smaller than those of *G. orientalis, B. clausii,* and *G. boraciitolerans* (4,335, 4,350, 4,441, and 4,510 genes, respectively), but larger than those of G*. halophilus, G. kekensis, G. massiliensis, B. alcalophilus, G. ureilyticus,* and *G. lacisalsi,* (2,999, 3,842, 3,887, 3,973, 4,066, and 4,290 genes, respectively). The gene distribution into COG categories was similar among all compared genomes (Figure 6). In addition, the AGIOS analysis showed that strain Marseille‐P2481 shared 2,103, 2,112, 2,004, 2,027, 1,461, 1,982, 1,695, 1,539, and 1,578 orthologous proteins with *G. lacisalsi, G. orientalis, G. massiliensis, G. kekensis, G. boraciitolerans, G. ureilyticus,* G*. halophilus, B. alcalophilus,* and *B. clausii*, respectively (Table 6). When comparing strain Marseille‐P2481 to other species, AGIOS values were 69.8, 70.0, 71.0, 71.8, 72.0, 72.1, and 72.3% with G*. halophilus, G. ureilyticus, G. boraciitolerans, G. kekensis, G. massiliensis, G. orientalis,* and *G. lacisalsi,* respectively (Table 6), but ranged from 62.9% to 64.5% with *B. clausii* and *B. alcalophilus*, respectively (Table 6). In addition, dDDH values relatedness of strain Marseille‐P2481 and the compared closest species varied between 19.1 and 28.67% and were 20.5, 19.8, 21.6, 20.1, 19.1, 21.4, 19.3, 23.6, and 28.67% for *G. lacisalsi, G. orientalis, G. massiliensis, G. kekensis, G. boraciitolerans, G. ureilyticus,* G*. halophilus, B. alcalophilus,* and *B. clausii,* respectively *(*Table 7). Finally, AAI values relatedness between strain Marseille‐P2481, *G. lacisalsi, G. orientalis, G. massiliensis, G. kekensis, G. boraciitolerans, G. ureilyticus,* and G*. halophilus* were 68.72, 68.19, 68.18, 67.90, 68.08, 64.69, and 64.37%, respectively, but were lower when compared with *B. alcalophilus* and *B. clausii*, with 51.72 and 50.73%, respectively (Table 8). These dDDH and AAI values were less than the 70% and 95–96% threshold values for species demarcation, respectively (Chun et al., 2018; Klappenbach et al., 2007; Meier‐Kolthoff, Auch, Klenk, & Göker, 2013; Richter & Rosselló‐Móra, 2009; Rodriguez‐R & Konstantinidis, 2014).

**Table 6:** Numbers of orthologous proteins (upper right) and AGIOS values (lower left, %) obtained between compared genomes. The numbers of proteins per genome are indicated in bold

	GT	GL	GO	GM	GK	GB	GU	GH	BA	BC
GT	**4,333**	2,103	2,112	2,004	2,027	1,461	1,982	1,695	1,539	1,578
GL	72.3	**4,268**	2,654	2,405	2,467	1,693	2,374	1,995	1,654	1,703
GO	72.1	85.2	**4,313**	2,370	2,412	1,686	2,318	1,940	1,656	1,710
GM	72.0	77.0	77.0	**3,839**	2,559	1,724	2,346	1,892	1,569	1,567
GK	71.8	76.6	76.7	88.7	**3,730**	1,724	2,345	1,907	1,596	1,594
GB	71.0	75.2	75.2	78.1	77.9	**4,587**	1,612	1,408	1,166	1,151
GU	70.0	72.6	72.6	72.6	72.6	71.5	**4,001**	1,880	1,605	1,599
GH	69.8	71.8	71.9	71.9	71.7	70.7	70.6	**3,156**	1,348	1,363
BA	64.5	65.6	65.4	65.6	65.6	64.7	65.1	65.1	**4,269**	1,532
BC	62.9	63.0	62.8	62.8	62.7	62.1	62.9	63.1	66.6	**4,449**

GT: *Gracilibacillus timonensis* Marseille‐P2481; GL: *Gracilibacillus lacisalsi* DSM 19029; GO: *Gracilibacillus orientalis* XH‐63; GM: *Gracilibacillus massiliensis* Awa‐1; GK: *Gracilibacillus kekensis* K170; GB: *Gracilibacillus boraciitolerans* JCM 21714; GU: *Gracilibacillus ureilyticus* MF38; GH: *Gracilibacillus halophilus* YIM‐C55.5; BA: *Bacillus alcalophilus* ATCC 27647; BC: *Bacillus clausii* KSM‐K16.

**Table 7:** dDDH values obtained by comparison of all studied genomes

	GL	GO	GM	GK	GB	GU	GH	BA	BC
GT	20.5% ± 2.35	19.8% ± 2.3	21.6% ± 2.35	20.1% ± 2.3	19.1% ± 2.3	21.4% ± 2.35	19.3% ± 2.3	23.6% ± 2.4	28.67% ± 2.4
GL		29.1% ± 2.4	21.0% ± 2.35	20.9% ± 2.35	20.2% ± 2.3	19.4% ± 2.3	18.7% ± 2.25	18.1% ± 2.25	24.4% ± 2.35
GO			21.0% ± 2.35	20.9% ± 2.35	19.9% ± 2.3	19.4% ± 2.25	18.2% ± 2.25	18.4% ± 2.25	25.2% ± 2.4
GM				35.4% ± 2.45	22.2% ± 2.35	19.4% ± 2.3	19.1% ± 2.3	19.9% ± 2.3	31.2% ± 2.5
GK					21.8% ± 2.35	19.7% ± 2.3	19.2% ± 2.3	18.4% ± 2.25	29.5% ± 2.45
GB						18.5% ± 2.25	17.4% ± 2.2	18.2% ± 2.25	33.9% ± 2.5
GU							16.9% ± 2.2	20.9% ± 2.3	24.6% ± 2.4
GH								27.2% ± 2.4	29.8% ± 2.45
BA									27.4% ± 2.45

GT: *Gracilibacillus timonensis* Marseille‐P2481; GL: *Gracilibacillus lacisalsi* DSM 19029; GO: *Gracilibacillus orientalis* XH‐63; GM: *Gracilibacillus massiliensis* Awa‐1; GK: *Gracilibacillus kekensis* K170; GB: *Gracilibacillus boraciitolerans* JCM 21714; GU: *Gracilibacillus ureilyticus* MF38; GH: *Gracilibacillus halophilus* YIM‐C55.5; BA: *Bacillus alcalophilus* ATCC 27647; BC: *Bacillus clausii* KSM‐K16.

**Table 8:** Average amino acid identity (AAI) values (%) between *Gracilibacillus timonensis* strain Marseille‐P2481T and other closely related species

	GL	GO	GM	GK	GB	GU	GH	BA	BC
GT	68.72	68.19	68.18	67.90	68.08	64.69	64.37	51.72	50.73
GL		85.64	77.21	76.84	75.47	70.41	68.82	52.40	51.31
GO			76.88	76.74	75.23	70.21	68.17	51.95	50.76
GM				90.32	79.78	70.72	68.09	52.02	50.74
GK					80.04	70.55	68.19	52.31	50.83
²GB						69.60	67.34	51.99	50.92
GU							67.03	52.53	51.16
GH								51.53	50.77
BA									57.85

GT: *Gracilibacillus timonensis* Marseille‐P2481; GL: *Gracilibacillus lacisalsi* DSM 19029; GO: *Gracilibacillus orientalis* XH‐63; GM: *Gracilibacillus massiliensis* Awa‐1; GK: *Gracilibacillus kekensis* K170; GB: *Gracilibacillus boraciitolerans* JCM 21714; GU: *Gracilibacillus ureilyticus* MF38; GH: *Gracilibacillus halophilus* YIM‐C55.5; BA: *Bacillus alcalophilus* ATCC 27647; BC: *Bacillus clausii* KSM‐K16.

## 4 DISCUSSION

Due to the concept of microbial culturomics, aiming at exploring the diversity of the human microbiota as exhaustively as possible, many new bacterial species have been discovered over the past 5 years (Lagier et al., 2016). This concept is based on the diversification of physicochemical parameters of culture conditions (Lagier et al., 2012, 2016; Lagier, Hugon, et al.,^2015^) to mimick as closely as possible the entirety of selective constraints that have shaped the human flora. To date, 329 new species have been characterized (Lagier et al., 2017). These new species include 52 species belonging to the order *Bacillales*, which is one of the most represented bacterial orders (Lagier et al., 2016). Using hypersaline conditions, many hitherto unknown bacteria extremely and or moderately halophilic have been identified in humans, including strain Marseille‐P2481. To the best of our knowledge, this is the first *Gracilibacillus* species described in the human gut. Whether it is a resident species of the human gut or a transitory species brought by food is as yet unknown. Its phenotypic, phylogenetic, and genomic characteristics suggested that it represents a new species within the genus *Gracilibacillus*. Members of this genus are generally Gram‐positive bacteria, aerobic, motile, moderately halophile and produce white colonies although *G. boraciitolerans* forms pink to red colonies (Ahmed, Yokota, & Fujiwara, 2007), and endospore‐forming. However, *Gracilibacillus timonensis* sp. nov. differs from other *Gracilibacillus* species in colony color and metabolism of β‐galactosidase, l‐arabinose, and d‐mannitol. In addition, its genomic DNA G + C content differed from those of other *Gracilibacillus* species, and the dDDH, AAI, and AGIOS values comforted its new species status.

## 5 CONCLUSION

The moderately halophilic strain Marseille‐P2481 was isolated from a stool sample of a 10‐year‐old healthy Senegalese boy as part of a study of halophilic bacteria from the human gut. Based on its phenotypic, phylogenetic, and genomic characteristics, this strain is proposed to represent a novel species in the genus *Gracilibacillus*, for which the name *Gracilibacillus timonensis* sp. nov. is proposed. Strain Marseille‐P2481^T^ is the type strain of *Gracilibacillus timonensis* sp. nov.

### 5.1 Description of *Gracilibacillus timonensis* sp. nov

*Gracilibacillus timonensis* (*ti.mo.nen*′*sis*, N. L adj. masc., *timonensis* of Timone, the name of the main hospital of Marseille, France, where the type strain was first isolated).

The bacterium is preferentially aerobic but is able to grow in anaerobic and microaerophilic atmospheres at 37°C. Strain Marseille‐P2481^T^ is able to grow in media containing up to 20% (w/v) NaCl, but no growth occurs in the absence of NaCl. The optimal culture conditions are 37°C, pH 7.0‐8.0, and 7.5% (w/v) NaCl. After 48 hr of incubation at 37°C on our home‐made culture medium (7.5% [w/v] NaCl), colonies are creamy orange and circular and have a mean diameter of 0.2 μm. Cells are Gram‐positive, motile rods (with peritrichous flagella) that form endospores rods and are slightly curved, with mean diameter and length of 0.5 and 1.9 μm, respectively.

Using an API ZYM strip, positive results were obtained for esterase, esterase lipase, acid phosphatase, naphtol‐AS‐BI‐phosphohydrolase β‐galactosidase, β‐glucosidase, and α‐glucosidase activities, but no reaction was observed for alkaline phosphatase, lipase, Leucine arylamidase, Valine arylamidase, Cystine arylamidase, α‐galactosidase, β‐glucuronidase, trypsin, α‐chymotrypsin, α‐mannosidase, α‐fucosidase, and N‐acetyl‐β‐glucosaminidase. The API 50CH strip revealed that strain Marseille‐P2481 exhibited esculin hydrolysis, but negative reactions were obtained for d‐arabitol, l‐arabitol, d‐glucose, d‐fructose, d‐fucose, d‐galactose, d‐lactose, d‐maltose, d‐ribose, d‐saccharose, d‐lyxose, d‐mannose l‐sorbose, d‐tagatose, d‐turanose, d‐xylose, l‐xylose, d‐arabinose, l‐arabinose, d‐sorbitol, d‐cellobiose, d‐melezitose, d‐melibiose, d‐trehalose, d‐raffinose, l‐rhamnose, d‐adonitol, d‐mannitol, l‐fucose, amygdalin, arbutin, erythritol, dulcitol, gentiobiose, glycerol, glycogen, inositol, inulin, salicin, starch, xylitol, αD‐glucopyranoside, methyl‐βD‐xylopyranoside, methyl‐αD‐mannopyranoside, potassium gluconate, potassium‐2‐ketogluconate potassium‐5‐ketogluconate, N‐acetylglucosamine. Using an API 20NE strip, fermentation of glucose, urease activity, and metabolism of l‐arginine, esculin and 4‐nitrophenyl‐βD‐galactopyrasinoside were positive. In contrast, nitrate and indole production, gelatinase activity and metabolism of d‐glucose, l‐arabinose, d‐mannose, d‐maltose, d‐mannitol, N‐acetyl‐glucosamine, potassium gluconate, capric acid, malic acid, trisodium citrate, and phenylacetic acid were negative. Cell membrane fatty acids are mainly saturated structures, with 12‐methyl‐tetradecanoic acid (45%) and hexadecanoic acid (16%) being the most abundant. No unsaturated structure was found. The genomic DNA G+C content is 39.8 mol%. The 16S rRNA and genome sequences are deposited in EMBL‐EBI under accession numbers LT223702 and FLKH00000000, respectively. The type strain of *Gracilibacillus timonensis* is strain Marseille‐P2481^T^ (= CSUR P2481 = DSM 103076).

## CONFLICT OF INTEREST

The authors declare no competing interest in relation to this research.
