# EXPRESSION OF CONCERN: Genome Sequence and Description of *Gracilibacillus timonensis* sp. nov. Strain Marseille‐P2481^T^, A Moderate Halophilic Bacterium Isolated from the Human Gut Microflora

**DOI:** 10.1002/mbo3.70241

**Published:** 2026-02-19

## Abstract

**EXPRESSION OF CONCERN:** A. Diop, E. Seck, G. Dubourg, N. Armstrong, C. Blanc‐Tailleur, D. Raoult, and P.‐E. Fournier, *“*Genome Sequence and Description of *Gracilibacillus timonensis* sp. nov. Strain Marseille‐P2481^T^, A Moderate Halophilic Bacterium Isolated from the Human Gut Microflora,” *MicrobiologyOpen* 8, no. 2 (2019): e00638, https://doi.org/10.1002/mbo3.638.

This Expression of Concern is for the above article, published online on 19 April 2018 in Wiley Online Library (wileyonlinelibrary.com), and has been issued by John Wiley & Sons Ltd. The Expression of Concern has been agreed due to questions raised about the study's adherence to French legal and ethical requirements for research involving human subjects, including questions regarding proper informed consent for research involving vulnerable populations. In addition, a third party has reported that Figure 4a in this article has strong similarities with a figure published in an earlier article by the same authors. The investigation into these concerns is ongoing. Therefore, the journal has decided to issue an Expression of Concern to inform and alert readers.

